# Supplementary material for: Membrane potential states gate synaptic consolidation in human neocortical tissue
Source: Nat Commun. 2024 Dec 12;15:10340. doi: 10.1038/s41467-024-53901-2 (PMC11638263; doi:10.1038/s41467-024-53901-2)
Supplement: Supplementary file 1 — Supplementary Information [file 41467_2024_53901_MOESM1_ESM.pdf]

Supplementary Information

Title

Membrane potential states gate synaptic consolidation in human neocortical tissue

Author list

Franz X. Mittermaier[1], Thilo Kalbhenn[2], Ran Xu[3], Julia Onken[3], Katharina Faust[3], Thomas Sauvigny[4], Ulrich W. Thomale[5], Angela M. Kaindl[6], Martin Holtkamp[7], Sabine Grosser[8], Pawel Fidzinski[9], Matthias Simon[2], Henrik Alle[1], Jörg R.P. Geiger[1]*

*Corresponding author. Email: joerg.geiger@charite.de. Address: Charitéplatz 1, 10117 Berlin, Germany

Affiliations

[1]Charité – Universitätsmedizin Berlin, corporate member of Freie Universität Berlin and Humboldt-Universität zu Berlin, Institute of Neurophysiology, Charitéplatz 1, 10117 Berlin, Germany

[2]University of Bielefeld Medical Center OWL, Department of Neurosurgery (Evangelisches Klinikum Bethel), Burgsteig 13, 33617 Bielefeld, Germany.

[3]Charité – Universitätsmedizin Berlin, corporate member of Freie Universität Berlin and Humboldt-Universität zu Berlin, Department of Neurosurgery, Charitéplatz 1, 10117 Berlin, Germany

[4]University Medical Center Hamburg-Eppendorf, Department of Neurosurgery, Martinistraße 52, 20246 Hamburg, Germany.

[5]Charité – Universitätsmedizin Berlin, corporate member of Freie Universität Berlin and Humboldt-Universität zu Berlin, Pediatric Neurosurgery, Augustenburger Platz 1, 13353 Berlin, Germany

[6]Charité – Universitätsmedizin Berlin, corporate member of Freie Universität Berlin and Humboldt-Universität zu Berlin, Department of Pediatric Neurology, Augustenburger Platz 1, 13353 Berlin, Germany

[7]Charité – Universitätsmedizin Berlin, corporate member of Freie Universität Berlin and Humboldt-Universität zu Berlin, Department of Neurology, Charitéplatz 1, 10117 Berlin, Germany

[8]Charité – Universitätsmedizin Berlin, corporate member of Freie Universität Berlin and Humboldt-Universität zu Berlin, Institute for Integrative Neuroanatomy, Charitéplatz 1, 10117 Berlin, Germany

[9]Charité – Universitätsmedizin Berlin, corporate member of Freie Universität Berlin and Humboldt-Universität zu Berlin, NeuroCure Cluster of Excellence, Neuroscience Clinical Research Center, Charitéplatz 1, 10117 Berlin, Germany

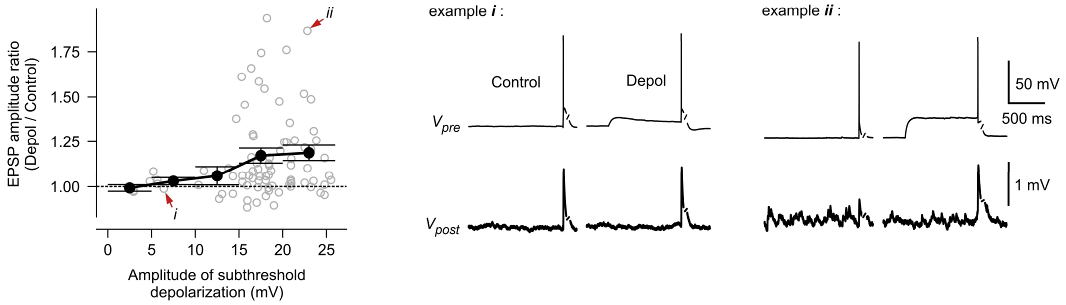

Supplementary Figure 1: Amplitudes of somatic subthreshold depolarizations had to be >10 mV to reliably cause enhancement of synaptic transmission. Left, scatterplot showing relative changes of excitatory postsynaptic potential (EPSP) amplitudes plotted against amplitudes of the subthreshold depolarizations. Experiments with 500 or 1000 ms 'Depol'-duration were pooled; n=83 paired recordings. Experiments with 'Depol' amplitudes <10 mV (n=7 paired recordings) were separately acquired and were excluded from other analyses. Black error bars correspond to mean ± s.e.m. of 5 mV bins. Arrows indicate two exemplary experiments, that are shown on the right. Right, two exemplary paired whole-cell recordings of synaptically connected pyramidal neurons. Somatic voltage traces of presynaptic neurons are shown in the top row and average postsynaptic EPSPs are shown in the bottom row.

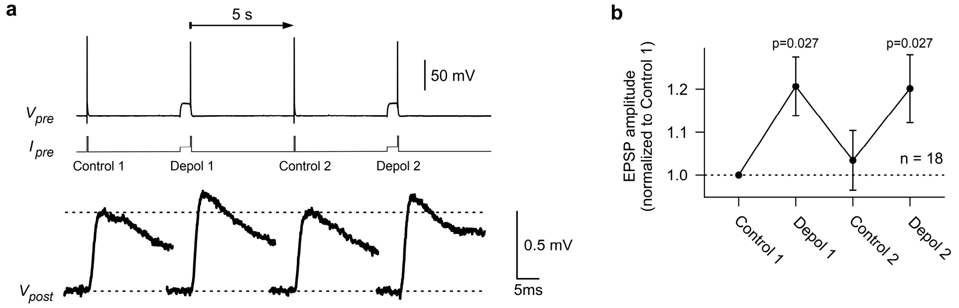

Supplementary Figure 2: Subthreshold modulation is a short-term effect. a, Exemplary paired recording of two synaptically connected pyramidal neurons, with somatic voltage trace of presynaptic neuron shown in the top row and average excitatory postsynaptic potentials (EPSP) shown in the bottom row. The stimulation protocol (I_{pre}) for this experiment was designed, so that 'Control' and 'Depol' conditions were triggered in an alternating manner, with an interval of 5 seconds. Note that the synaptic enhancement caused by the subthreshold depolarization ('Depol 1') was gone after the 5 second interval ('Control 2'), indicating a short-term effect. **b**, Summary plot of n = 18 paired recordings (these n=18 paired recordings were performed at 1.2 mM extracellular calcium concentration; two-sided Wilcoxon signed-rank tests; error bars show mean ± s.e.m).

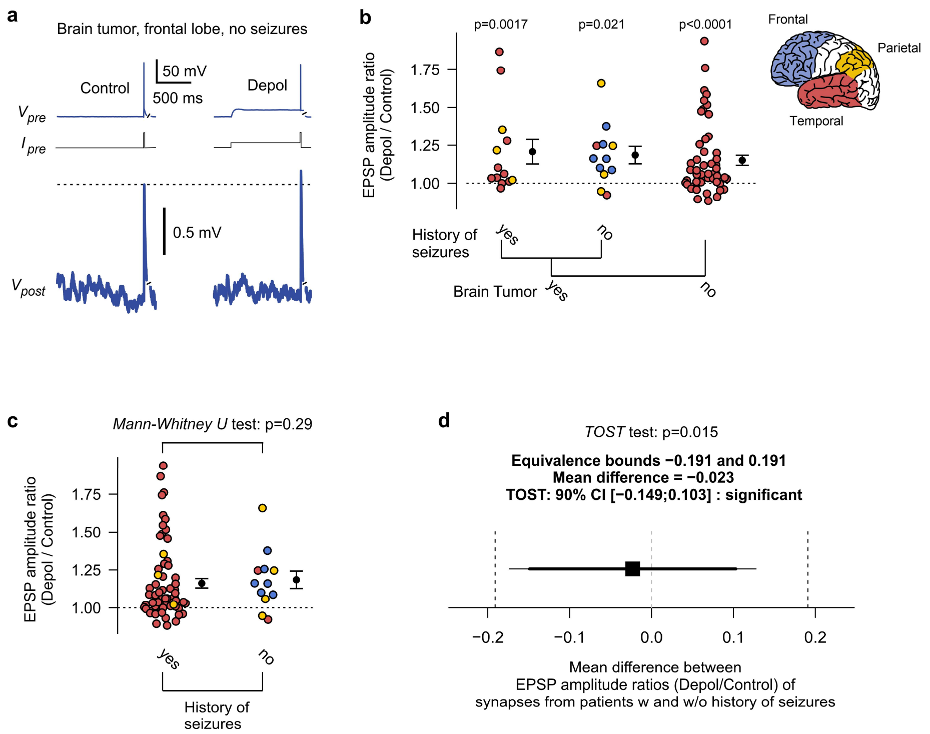

Supplementary Figure 3: Subthreshold modulation across region and disease. a, Exemplary paired whole-cell patch-clamp recording of synaptically connected pyramidal neurons, performed in cortical tissue from a patient with a brain-tumor in the frontal lobe, who had no documented seizures. Somatic voltage traces of presynaptic neuron are shown in the top row and average excitatory postsynaptic potentials (EPSP) are shown in the bottom row. **b**, Relative changes in EPSP amplitude plotted against disease parameters, namely whether experiments were performed in samples from patients with- or without brain tumors and with- or without a history of seizures (each data point is a single paired recording; experiments with 500 and 1000 ms ‘Depol’-length were pooled; n=76 paired recordings; patients who had no brain tumors underwent surgery for refractory epilepsy; two-sided Wilcoxon signed-rank tests; error bars indicate mean ± s.e.m). **c**, Data plotted analogously to panel b, but only separated into two groups based on ‘history of seizures’ (n=76 paired recordings; two-sided Mann-Whitney *U* test; error bars show mean ± s.e.m). **d**, Summary graph, depicting the result of a *TOST*-test procedure (*TOST* stands for two-one-sided-tests). The *TOST*-test is an equivalence test with the H_0, that the mean effects in two groups are not equivalent with regard to some equivalence bounds. We tested whether mean effects from patients with- or without a history of seizures are ‘practically equivalent’ and used equivalence bounds of ±0.8*s.d. (s.d. is the standard deviation of the pooled data; as a rule of thumb, effect sizes >0.8*s.d. are considered large[1]). We rejected the H_0 and conclude that the mean effects in the two groups are ‘practically equivalent’.

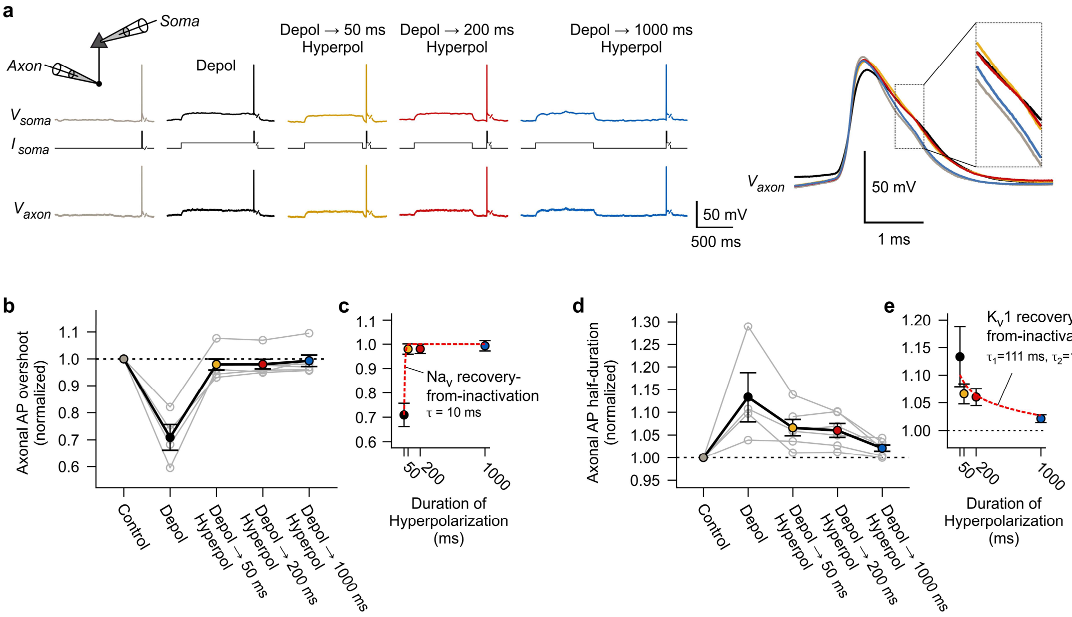

Supplementary Figure 4: Subthreshold hyperpolarizations of varying durations in sequences of de- and hyperpolarizations. **a**, Exemplary paired somato-axonal recording. Following 800 ms long depolarizations, hyperpolarizations of 50, 200 and 1000 ms duration were induced before eliciting action potentials (AP) by somatic current injection. The shape of the resulting axonal APs was investigated. Right, overlay of the axonal APs for the different conditions. **b**, Relative changes of AP overshoot amplitude plotted across the different stimulation conditions (n=6 somato-axonal recordings; error bars indicate mean ± s.e.m.). Note, 50 ms hyperpolarizations were already sufficient to restore the overshoot amplitude. **c**, duration of hyperpolarizations plotted on the x-axis and relative change of AP overshoot on the y-axis. Red dashed line is a mono-exponential function with a time-constant of 10 ms reflecting the reported time constant for the recovery from inactivation of voltage gated sodium channels in nucleated patches of human layer 2 & 3 pyramidal neurons[2]. **d-e**, same as panels b-c, but for AP half-duration and recovery-from-inactivation of axonal K_V1 voltage gated potassium channels (see main Fig. 2d).

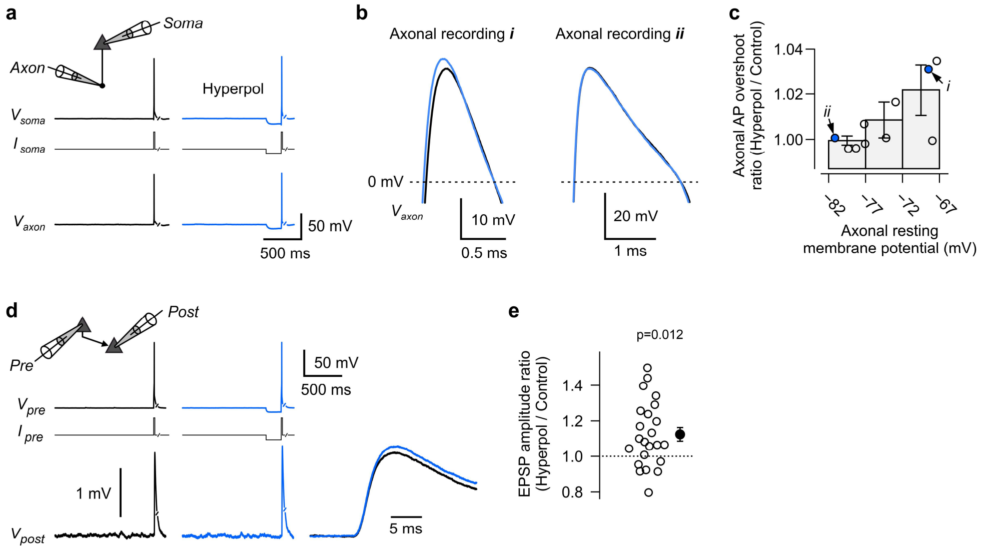

Supplementary Figure 5: Isolated somatic hyperpolarizations (without preceding depolarizations) modulate axonal action potential (AP) amplitudes in axons with more depolarized resting membrane potentials and modestly enhance synaptic transmission. **a**, Somato-axonal recording. APs were elicited in the soma from resting membrane potential or following an isolated hyperpolarization ('Hyperpol' condition). **b**, Two exemplary recordings of axonal APs are shown (*i* & *ii*). **c**, Scatterplot showing resting membrane potential of axons on the x-axis and relative changes in AP overshoot amplitudes on the y-axis (n=10 somato-axonal recordings). Bars correspond to 5 mV-bins (error bars indicate mean ± s.e.m.). The arrows indicate the example experiments shown in panel b. **d**, Paired recording of synaptically connected pyramidal neurons. Postsynaptic traces were averaged over multiple trials. **e**, Summary graph showing relative changes in excitatory postsynaptic potential (EPSP) amplitude in response to the 'Hyperpol' condition (n=23 paired recordings; two-sided Wilcoxon signed-rank test; error bar indicates mean ± s.e.m.).

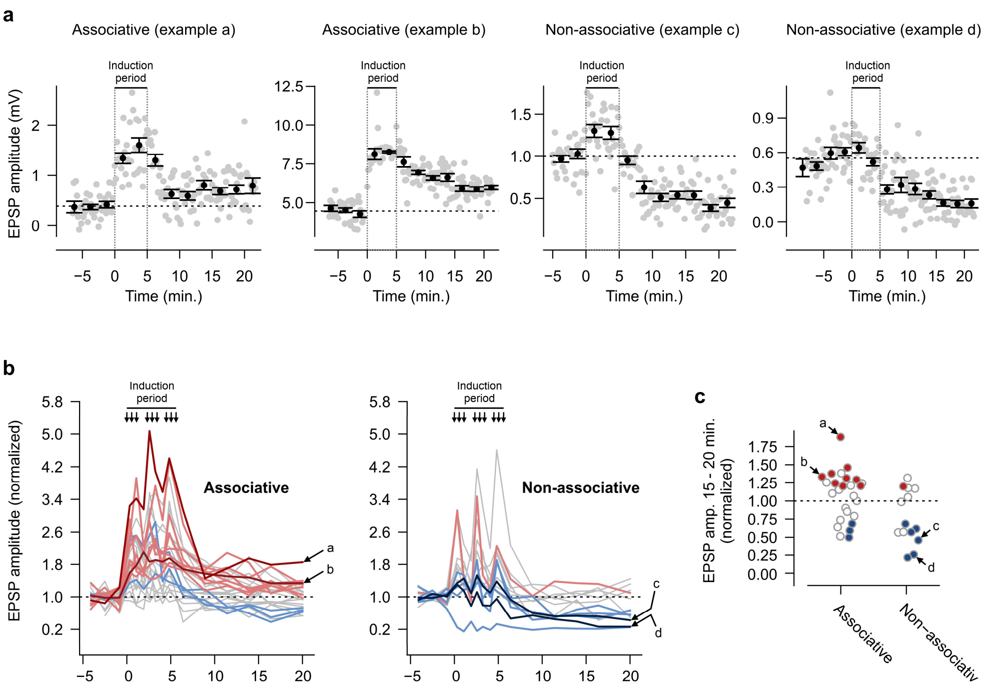

Supplementary Figure 6: Response of synapses to long-term plasticity induction is variable and can be strong. a, Single-trial excitatory postsynaptic potential (EPSP) amplitude plotted over time for four selected synapses recorded in paired whole-cell configuration (black error bars indicate mean ± s.e.m of 2.5-minute bins). Time point '0' marks start of plasticity induction (see Fig. 4e). Note the clear potentiation and depression in response to 'Associative' and 'Non-associative' paradigms, respectively. **b**, Normalized EPSP amplitude plotted over time for all experiments in the data set (each line corresponds to a single paired recording of synaptically connected neurons; n=27 paired recordings for 'Associative' stimulation paradigm, n=14 paired recordings for 'Non-associative' stimulation paradigm). Two ARIMA models, one accounting for and the other one not accounting for plasticity induction with a step variable, were fit to the time series data of individual experiments. Experiments in panel b-c are shown in color if the model, which accounted for plasticity induction, fit the data better (determined by Akaike information criterion), indicating a relevant change in synaptic amplitude. The four example experiments shown in panel a are indicated by a darker shade and arrows. **c**, Summary graph showing effect 15-20 min. after start of induction in response to 'Associative' (n=27) and 'Non-associative' (n=14) paradigms (each data point is one synapse; normalized to pre-induction period).

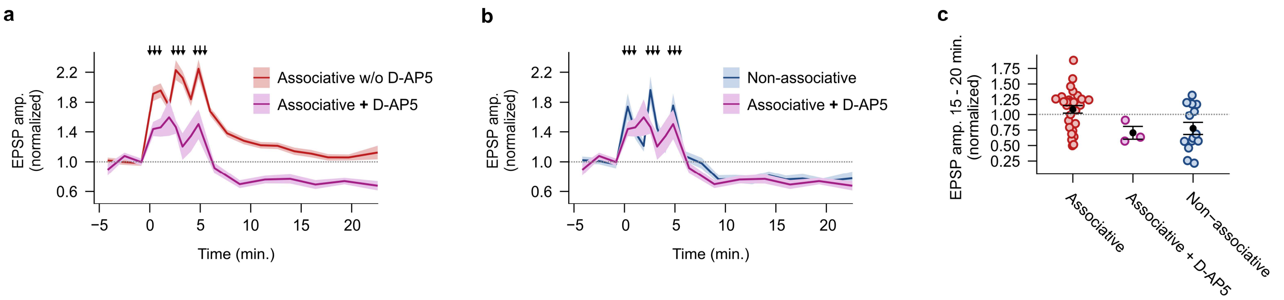

Supplementary Figure 7: D-AP5 converts associative stabilization of synapses to lasting depression. a, Mean excitatory postsynaptic potential (EPSP) amplitudes plotted over time for all experiments with 'Associative' plasticity induction. Purple line corresponds to experiments in presence of 50 µM D-AP5 (n=3 paired recordings) to block NMDA-receptors (normalized to pre-induction period; mean ± s.e.m). **b,** Experiments with 'Associative' paradigm but in presence of D-AP5 are shown in one plot with experiments with 'Non-associative' paradigm. **c,** Summary graph showing long-term plasticity effect in response to the different experimental conditions (each data point is one synapse; amplitudes were normalized to pre-induction period; error bars indicate mean ± s.e.m).

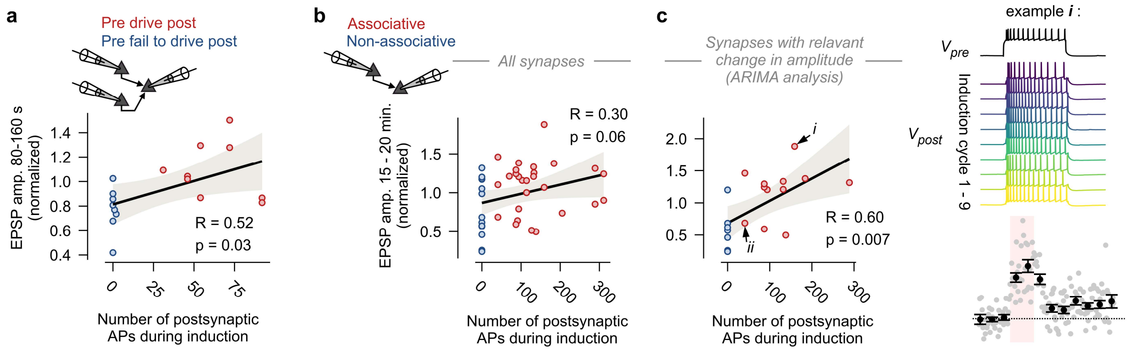

Supplementary Figure 8: The magnitude of associative plasticity depends on the number of postsynaptic action potentials (AP) elicited during the induction period. a, ‘Pre drive post’ and ‘Pre fail to drive post’ experimental paradigms (see main Fig. 5b-e and main text for description of the paradigms). Scatterplot showing the number of postsynaptic action potentials (AP) elicited during the induction period of these experiments on the x-axis and the excitatory postsynaptic potential (EPSP) amplitude of the post-induction period (normalized to the pre-induction EPSP amplitude) on the y-axis. The black line corresponds to a regression line and the filled area to 95% confidence intervals (c.i.) of a linear model. The Pearson correlation coefficient and the p-value are shown as insets (n=17 synapses; Correlation test based on t-statistic). **b**, same as panel a but for the ‘Associative’ and ‘Non-associative’ experimental paradigms (see main Fig. 5f-h and main text for description of the paradigms; n=41 synapses; Correlation test based on t-statistic). **c**, same as panel b but only for those synapses that displayed a relevant change in EPSP amplitude following plasticity induction (ARIMA time-series models; see Supplementary Fig. 6b-c; see Methods; n=19 synapses; Correlation test based on t-statistic). Arrows indicate two example experiments that are depicted on the right. Right, induction periods of two example recordings. Top rows show presynaptic voltage traces for one of the nine induction cycles. Bottom rows show postsynaptic voltage traces for the nine induction cycles. Summary plots below the traces show EPSP amplitude over time. The light red background indicates the induction period.

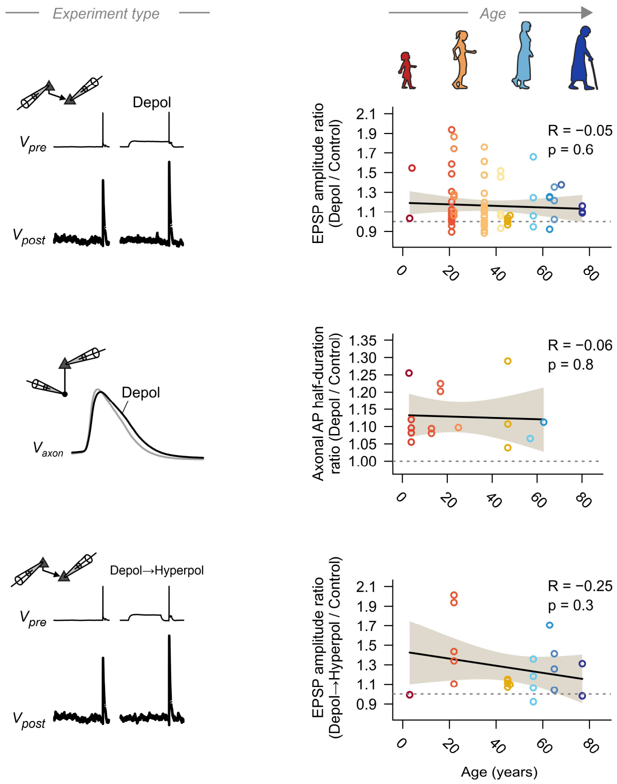

Supplementary Figure 9: **Lack of evidence for age-dependency of the studied effects**. Left column shows experiment type for each row. Right column displays scatterplots showing datapoints of the respective experiments across age. Pearson correlation coefficients (R) and p-values (Correlation test based on t-statistic) are shown as insets. The black lines correspond to regression lines and the filled areas to 95% confidence intervals of linear models. Top row: n=76 paired recordings from n=16 patients; middle row: n=15 somato-axonal recordings from n=9 patients; bottom row: n=21 paired recordings from n=8 patients.

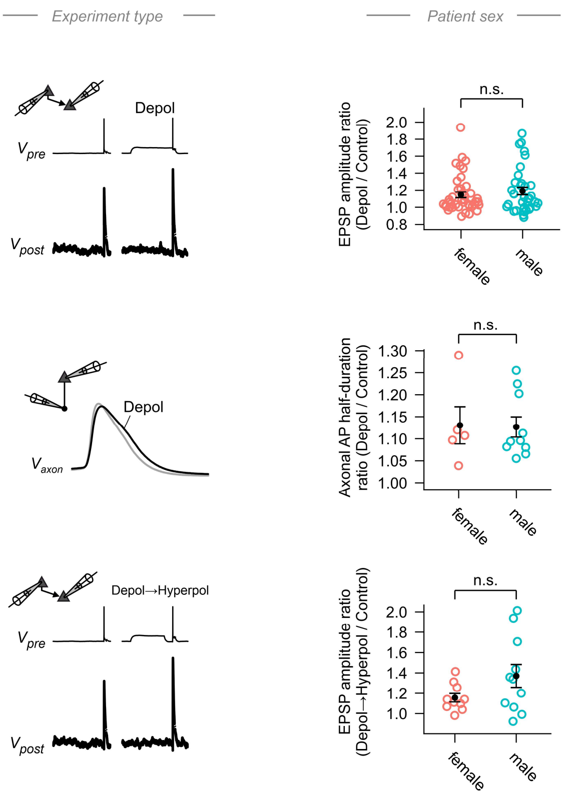

Supplementary Figure 10: **Studied effects disaggregated by patient sex**. Left column shows experiment type for each row. Right column shows datapoints of the respective experiments disaggregated by patient sex (error bars correspond to mean ± s.e.m.). n.s., not significant (two-sided Mann-Whitney *U* tests). Top row: n=76 paired recordings from n=16 patients; middle row: n=15 somato-axonal recordings from n=9 patients; bottom row: n=21 paired recordings from n=8 patients.

Supplementary references:

1 Cohen, J. *Statistical Power Analysis For The Behavioral Sciences*. 2nd edn, (Routledge, New York, 1988).

2 Wilbers, R. *et al.* Human voltage-gated Na(+) and K(+) channel properties underlie sustained fast AP signaling. *Sci Adv* **9**, eade3300 (2023). https://doi.org:10.1126/sciadv.ade3300
